# Phase Diagrams of Smart Copolymers Poly(N-isopropylacrylamide) and Poly(sodium acrylate)

**DOI:** 10.1155/2014/516076

**Published:** 2014-08-14

**Authors:** Iwona Zarzyka, Maria Laura Di Lorenzo, Marek Pyda

**Affiliations:** ^1^Department of Chemistry, Rzeszow University of Technology, 35-959 Rzeszow, Poland; ^2^Consiglio Nazionale delle Ricerche, Istituto di Chimica e Tecnologia dei Polimeri, c/o Compresorio Olivetti, Via Campi Flegrei 34, 80078 Pozzuoli, Italy; ^3^ATHAS-MP Company, Knoxville, TN 37922, USA

## Abstract

The phase behavior of linear poly(N-isopropylacrylamide) (PNIPA), linear copolymer poly(N-isopropylacrylamide) and poly(sodium acrylate) (PNIPA-SA), and chemically cross-linked PNIPA in water has been determined by temperature modulated differential scanning calorimetry (TM-DSC). Experiments related to linear polymers (PNIPA and PNIPA-SA) indicated nontypical demixing/mixing behavior with a lower critical solution temperature (LCST), which do not correspond to the three classical types of limiting critical behavior. Some similarities and differences are observed in comparison to our literature data using standard TM-DSC for PNIPA/water. Furthermore no influence of composition cross-linked PNIPA/water system on demixing/mixing temperature was observed.

## 1. Introduction

Nowadays stimuli-sensitive polymers, also called smart polymers, are the focus of our attention. These polymers give their own responses to external stimuli such as temperature, pH, electric field, light, biochemical compounds, or specific ions. Scientists attach a lot of importance to these properties as the key to technological development. The features could be adapted precisely to the demands of any user. The polymers find applications in many industrial areas. For this reason, smart polymers are a part of devices, both nanodevices and microdevices, used in medication's delivery, tissue engineering, bioseparation, sensors, and actuators and even artificial muscles [1–5]. Intelligent polymers are known for their responses to small variations, such as the external stimuli mentioned above. These variations result in both on the molecular scale (changes of properties of the surface either in hydrophilic or hydrophobic) and on the macroscopic scale. These include precipitation (linear polymers), a volume change transition (cross-linked polymers), and a change in the water content (hydrogels) [6].

Poly(N-isopropylacrylamide) (PNIPA) is representative for many stimuli-sensitive polymers as well as hydrogels. PNIPA characterizes the discontinuous volume transition [7].

The most important aspect of PNIPA is its phase transition from a hydrophilic to a hydrophobic state at the LCST [8, 9]. These changes are connected to temperature, so above LCST there are hydrophobic interactions between polymer chains that entail participation or deswelling. Below LCST the swelling or solubility and the separation of the polymer chains rise and PNIPA is more hydrophilic [10, 11].

The behavior of PNIPA depends on structure of polymers, especially on the kind of comonomer and copolymer [12–18], as well as the character of end groups [19–23].

Solubility of linear PNIPA has a minimum of about 45 wt% polymer in dependence on molecular weight of polymer at temperature 26-27°C [24] or under 25°C according to [25].

The demixing/mixing of water soluble polymers from their solution can be subdivided into three types. The type of demixing is related to a specific swelling behavior of the corresponding chemical network [26–28]. These three types of phase behavior are described in detail and are schematically illustrated in [28]. They are explained by the theory developed by Flory [29], Huggins [30], and Staverman and Van Santen [31].

Afroze et al. studied the influence of molecular weight and concentration of polymer on the demixing temperature [24]. They indicated the type II demixing behavior of PNIPA/water. A limiting critical situation at infinite molar mass at off-zero concentration is around 50 wt% polymer [32].

A type II LCST demixing behavior of PNIPA was confirmed by investigations of van Mele group [33].

The LCST can be measured by turbidimetry or ultraviolet/visible (UV/VIS) spectroscopy, dynamic light scattering, differential scanning calorimetry (DSC), small-angle neutron scattering (SANS), and other techniques [34].

In our work TM-DSC was used to determine a phase diagram for linear PNIPA, linear copolymer PNIPA-SA, and cross-linked PNIPA (PNIPA-BIS)/water system. The phase diagram of PNIPA was shown many times by other scientists, but this time a somewhat different shape of the curve was found. Next, the phase diagram of PNIPA-SA was constructed and compared with the curve of PNIPA without SA. Copolymer PNIPA-SA is known and investigated earlier [35–39]. An introduction of SA increases water absorption capacity of PNIPA. The gel swelling properties were measured as a function of pH, ionic strength, temperature, salt presence, and other stimuli [40–42], but none have shown the completed phase diagram of this copolymer. Preliminarily, only results for the range of 10–35% wt gels PNIPA-SA were presented in the paper [43].

## 2. Experimental Part

### 2.1. Materials

N-Isopropylacrylamide (NIPA) 97%, N,N,N′,N′-tetramethylethylenediamine (TEMED) 99%, and sodium acrylate (SA) 97% were purchased from Sigma-Aldrich. N,N′-methylenebisacrylamide (BIS) ≥ 99.5% was provided by Fluka. Ammonium persulfate (APS) 98% was obtained from Riedel-de Haën. All chemicals were used as received.

### 2.2. Hydrogel Synthesis

PNIPA gels were prepared by free radical polymerization in water, which is a solvent for all components of the mixture [7, 43]. The preparation of the linear copolymer PNIPA-SA and cross-linked copolymer PNIPA-BIS was similar but in the case of this later one, (the cross-linking degree, defined as the ratio of moles of cross-linking agent and the moles of repeating unit, equals 1,47%).

The molar weight distribution of the linear polymer samples was investigated by the gel permeation chromatography (GPC) with N,N′-dimethylformamide as the solvent. The molar weight results for the three polymer samples are shown in Table 1.

### 2.3. Preparation of Polymer/Water Mixtures

Gels were dried under vacuum for a few days at 70°C, after which the water content was less than 1.5 wt%, as determined by thermogravimetric analysis. Polymer/water mixtures containing from 5 to 90% of water were prepared by direct addition of the appropriate amount of water to the dried gels and then stored in a refrigerator overnight. The homogeneous mixtures were encapsulated in aluminum hermetic pans for calorimetric analysis. Simultaneously, sample weight loss was measured by TGA to check the preparation procedure and determine the effective water content (error ≤ 1 wt%).

### 2.4. Thermal Analysis

#### 2.4.1. Thermogravimetry

Water content of the hydrogels was determined with a Perkin Elmer Pyris Diamond TG/DT analyzer. Samples were placed in aluminum open sample pans (capacity 90 *μ*L) and heated at 20°C/min from room temperature to complete evaporation of water. High purity nitrogen gas was fluxed into the furnace at a flow rate of 50 mL/min. As mentioned above, thermogravimetry was used to check the water content of the hydrogels, simultaneously with DSC analyses, as well as to determine eventual residual water in the dried polymers.

#### 2.4.2. Calorimetry

MTDSC measurements were performed on a TA Instruments Q1000 DSC with the modulated DSC option and an RCS cooling accessory. High purity nitrogen gas was fluxed at 50 mL/min during all measurements and thermal treatments. Indium and sapphire were used for temperature, heat of fusion, and heat capacity calibration, respectively. Samples (swollen gels) of 1-2 mg were placed into Tzero hermetic aluminum pans. The standard temperature modulation DSC conditions were used with an amplitude modulation of *A*_*T*_ = 0.8°C and a period of* p* = 60 s at an underlying heating rate of 5°C min^−1^. The measurements were carried out from −50 to 80°C. Samples were cooled inside of DSC cell at a rate of 10 K/min using standard DSC method. No noticeable difference in the sample mass before and after each DSC run was detected, thus indicating that no water evaporation took place during DSC analysis using the Tzero hermetic aluminum pans.

## 3. Results and Discussion

For the obtained polymers-linear PNIPA, linear copolymer PNIPA-SA (gel contains 5 wt% of sodium acrylate in the dry state), and cross-linked PNIPA (PNIPA-BIS), the influence of hydrogel content on the LCST is analyzed as function of water swelling in the range from 10 to 95 wt% of gel using DSC and TM-DSC.

**Figure 1:**
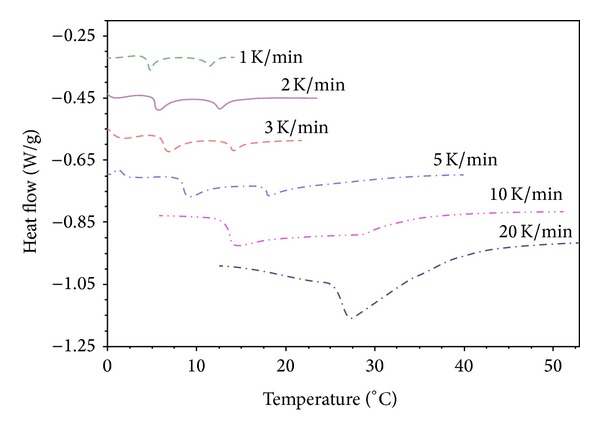
DSC thermograms of PNIPA-SA/water (20%/80%) system in equilibrium swelling degree. Heating was performed with 1, 2, 3, 5, 10, and 20 K/min after cooling with 10 K/min.

As was illustrated in Figure 1 the “phase separation” point determined by DSC depends on the temperature heating rate.

**Figure 2:**
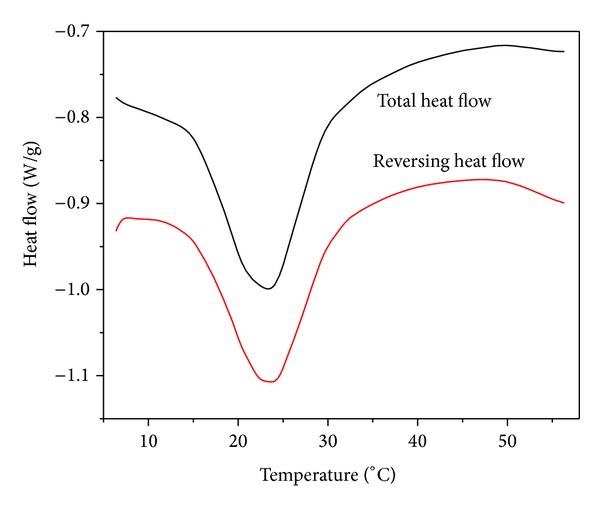
TM-DSC thermograms of PNIPA-SA/water (20%/80%) system in equilibrium swelling degree, upon heating rate of 5 K/min and cooling rate of 10 K/min.

TM-DSC measurements of the same sample have given somewhat different results (Figure 2). Only one peak has been noticed both on curve of total heat flow and on curve of reversing heat flow in contradistinction to DSC curve despite the same heating rate (5 K/min) (Figures 1 and 2). Figure 2 shows a comparison of total and reversing heat flow of demixing where endotherms are similar and the process is reversing. The demixing temperature (LCST) was estimated as onset temperature.

**Figure 3:**
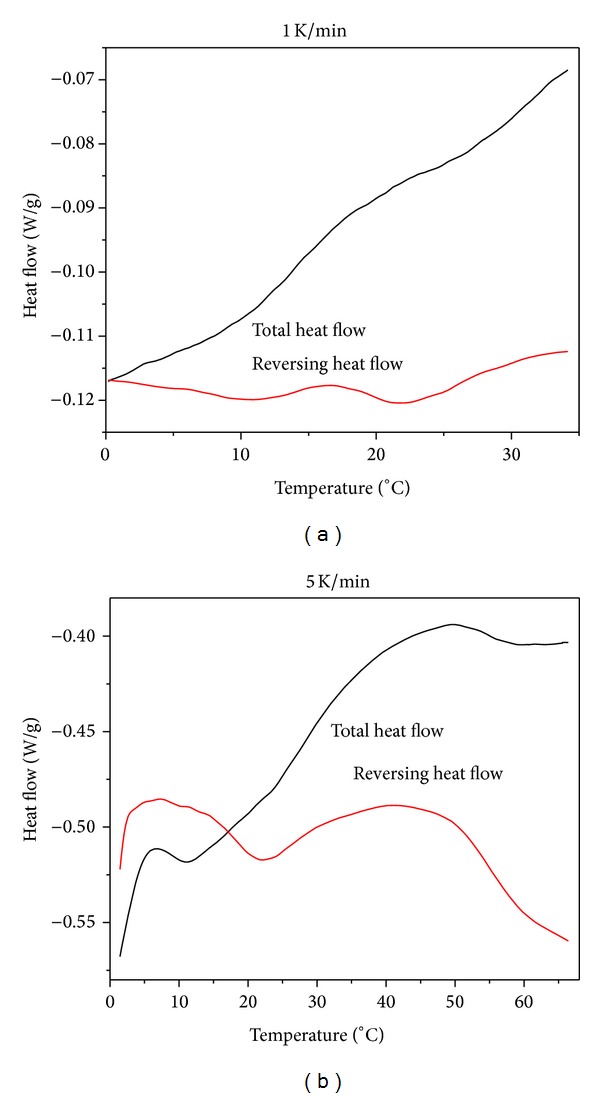
TM-DSC thermograms of PNIPA-SA/water (40%/60%) system in equilibrium swelling degree, upon heating rate of (a) 1 K/min and (b) 5 K/min and cooling rate of 10 K/min.

Due to the fact that this work was mostly qualitative analysis of phase separation of PNIPA and its comonomers/water systems, all measurements were performed at 5 K/min heating rate. This heating rate was selected based on the further preliminary investigations which showed the most convenient heating rate to such type of analysis (Figure 3).

**Figure 4:**
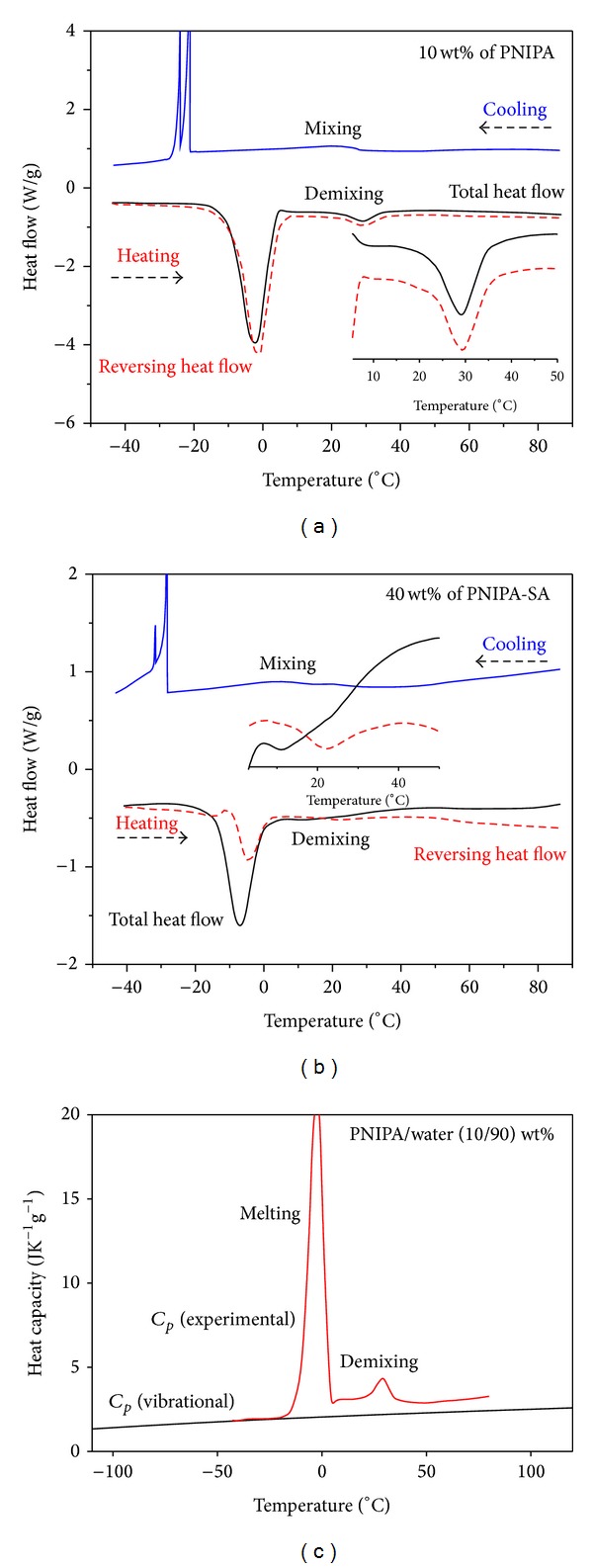
((a), (b)) TM-DSC thermogram of gel/water system in equilibrium swelling degree: (a) PNIPA/water (10%/90%) and (b) PNIPA-SA (40%/60%). Onset values of LCST were taken from the TM-DSC plots (reversing heat flow) upon first and second heating at 5°C/min. Cooling was performed with 10 K/min by standard DSC. Endotherms around 0°C on heating and exotherms around −30°C on cooling are related to melting and crystallization of water, respectively. (c) Experimental, reversing heat capacity of the 10%/90% PNIPA/water and their vibrational baseline, *C*_*p*_ (vibrational) [44, 45].

For better results, the heating rate should be as slow as possible. Figures 4(a) and 4(b) show two examples of results of reversing heat flow and total heat flow from TM-DSC measurements for demixing/mixing process of polymer/water system: PINIPA (10% : 90%) (Figure 4(a)) and PNIPA-SA (40% : 60%) (Figure 4(b)).

Next, Figure 4(c) shows an example of reversing heat capacity for the raw data presented in Figure 4(a). The reversing heat flow of PNIPA/water (10%/90%) was converted to reversing heat capacity *C*_*p*_ (experimental) and compared with vibrational heat capacity *C*_*p*_ (vibrational) of PNIPA/water system. Experimental and calculated, vibrational *C*_*p*_, data were in good agreement on a short range of temperature between −35 and −20°C. The only contribution to experimental heat capacity from below melting and demixing endotherms is given from vibration motion of PNIPA macromolecules and molecules of water. The solid, vibrational heat capacity, *C*_*p*_ (vibrational) (see Figure 4(c)), of 10/90 wt% PNIPA/water was estimated as a linear combination of the vibrational heat capacity of PNIPA, *C*_*p*_^*p*^ (vib), and water, *C*_*p*_^*w*^ (vib), listed as follows: *C*_*p*_ (vibrational) = *f*_*p*_ *C*_*p*_^*p*^ (vib) + *f*_*w*_ *C*_*p*_^*w*^ (vib), where *f*_*p*_ and *f*_*w*_ are the weight fractions of polymer and water, respectively. This vibrational heat capacity is a line that together with liquid heat capacity can construct a baseline which possibly separates a true heat capacity from excess heat or latent heat of melting and demixing processes of PNIPA/water system. Similar separation was performed for poly(vinyl methyl ether)/water in [46]. Full description of vibrational and liquid heat capacity PNIPA and PNIPA/water, including table of vibration spectrum, is presented in the other papers which are under review and preparation [44, 45]. Other interpretations of behaviors of apparent heat capacity PNIPA/water, in terms of hydrophilic and hydrophobic interactions between PNIPAM and the surrounding water molecules, are out of scope from TMDSC method.

**Figure 5:**
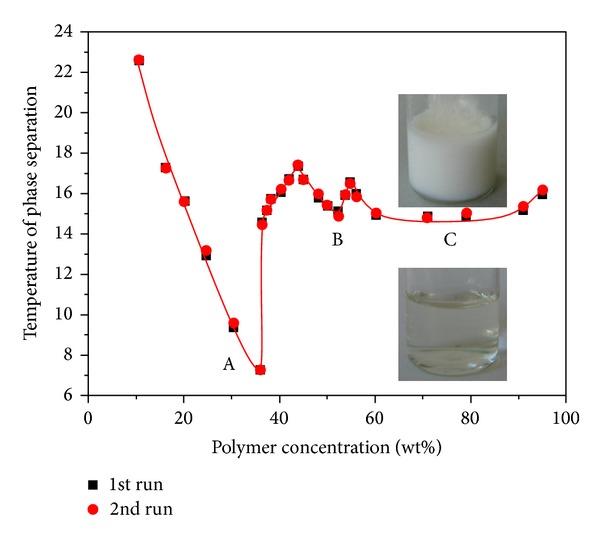
Phase diagram of linear PNIPA/water system; onset values of LCST were taken from the TM-DSC plots (reversing heat flow) upon first and second heating at 5°C/min; lines are guides for the eye.

**Figure 6:**
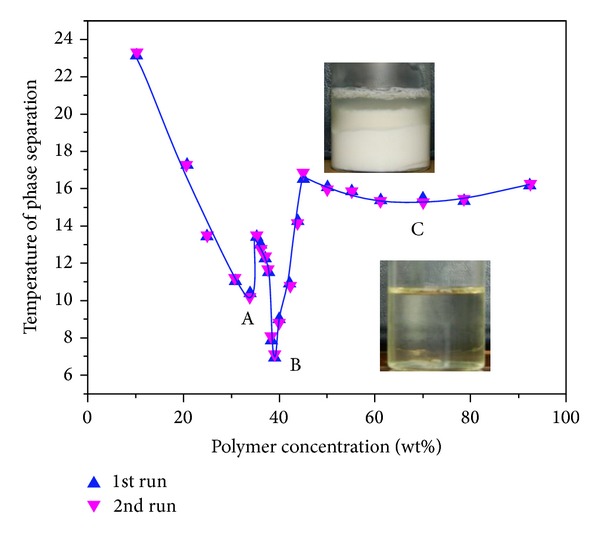
Phase diagram of linear copolymer PNIPA-SA/water system; onset values of LCST were taken from the TM-DSC (reversing heat flow) plots upon first and second heating at 5°C/min; lines are guides for the eye.

**Figure 7:**
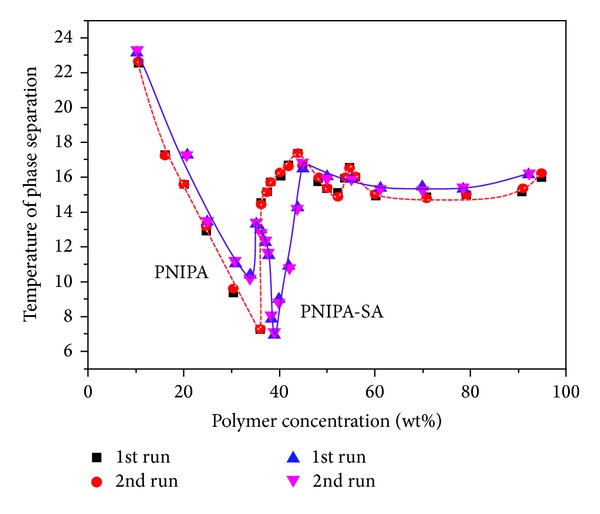
A comparison of phase diagrams of PNIPA and PNIPA-SA/water systems.

**Figure 8:**
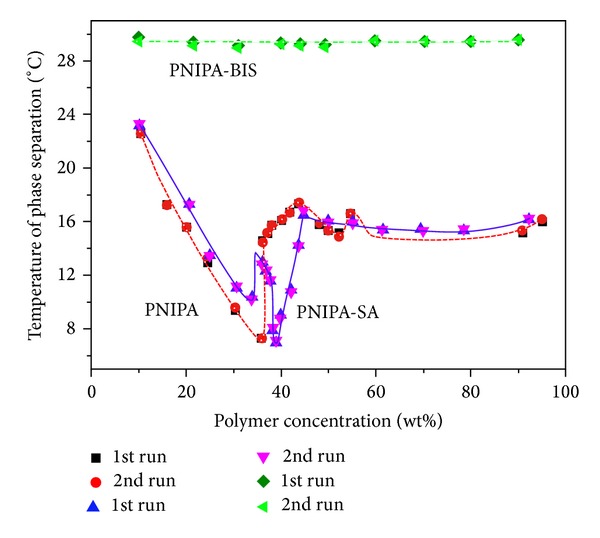
Phase diagrams of PNIPA, PNIPA-SA, and PNIPA-BIS/water systems; onset values of LCST were taken from the TM-DSC plots (reversing heat flow) upon first and second heating at 5°C/min.

In this paper, the measured values of LCST are plotted in Figures 5, 6, 7, and 8 as function of gel content. Three distinct two-phase areas are observed in case of PNIPA and PNIPA-SA.

Three critical points (A, B, and C) appear at around 36, 52, and 70 wt% hydrogel defined at approximately 7, 15.1, and 14.9°C, respectively, and are observed on the phase diagram of PNIPA in Figure 5. Two first two-phase areas (A and B) have clearly marked extrema in comparison to the third area (C) which has rather a plateau. Also, in Figure 5 two intersections between these three regions at around 44 and 55 wt% gel were defined at 17.3 and 16.65°C, where three phases coexist. These LCST values are repeated in the subsequent heating as we can see in Figure 5. Phase diagrams drawn on the basis of 1st and 2nd heating are congruous.

There was only one limiting critical concentration at approximately 40 wt% gel, observed at a temperature of about 23°C, so van Durme and coworkers [33] classified it like II type demixing behavior. Our investigation shows the phase diagram as a type III demixing behavior. There are two off-zero limiting critical concentrations [28] but in addition there is also a plateau.

The resulting phase diagram of the PNIPA/water system obtained in this paper is similar in a low concentration of polymer and different in high range compared to the phase diagram obtained for this system by van Durme and coworkers in [33]. In Figure 4(a) differences start above 36 wt% PNIPA. One of the differences is the high range of PNIPA concentration in PNIPA/water system which can be due to different placing of water by polymeric material. In our case it was by mixing and adding water to PNIPA while for van Durme and coworkers [33] it was prepared by evaporation of water. Two different critical points (B and C) can be a result of an unexpected morphology of polymer leading to a specific combination of hydrophilic and hydrophobic interactions between PNIPAM and water molecules.

A similar phase diagram is gained during measurements of LCST of copolymer PNIPA-SA gel/water system (Figure 6) for first (1st run) and second (2nd run) heating.

On the phase diagram (1st run) three minima (A, B, and C) at about 34, 39, and 70 wt% are present, which correspond to the temperatures 10, 7, and 15.4°C, respectively. As shown in Figure 6, the third area (C) of phase diagram has the plateau too. The intersections appear at about 35 and 45 wt%, appropriately at temperatures 13.4 and 16.5°C, respectively. In subsequent heating (2nd run), similar results were produced as shown in Figure 6.

Comparing the shapes of both phase diagrams PNIPA and PNIPA-SA/water system we can see some similarities and differences (Figure 7).

The first critical point on both curves is situated quite similarly, although an introduction of 5 wt% of SA into structure of PNIPA causes somewhat of an earlier occurrence of the critical point at 34 wt% gel and at a slightly higher temperature of 10°C, in comparison to the first minimum critical point of PNIPA/water system located at 36 wt% and 7°C (Figure 7).

Definitely larger changes are observed in case of second minimum of the limiting critical concentration. On the phase diagram of PNIPA-SA/water system, the second minimum of critical point is shifted from 52 wt% of PNIPA to 39 wt% of PNIPA-SA. Furthermore there is a visible change of the minimum in temperature with a decrease from 15.1 to 7°C.

The plateau of both phase diagrams is very similar (Figure 7). It should be noticed that the intersections are located in different gel content, but the distance between them is almost identical. The intersections on phase diagram of PNIPA-SA/water system appear to be about 10 wt% of gel content less than in case of PNIPA/water system. The second intersection of PNIPA-SA/water system (45 wt%, 16.5°C) nearly overlaps to the first intersection of PNIPA (44 wt%, 17.3°C).

The shape of the phase diagram of cross-linked PNIPA-BIS is completely different (Figure 8). Present is almost a straight line at the level of temperature 29.5°C. We can observe no dependence of the LCST on gel content in system PNIPA-BIS/water system.

## 4. Conclusions

In summary, the presented study indicated nontypical demixing behavior of PNIPA and PNIPA-SA. The phase diagram of PNIPA/water system determined that basis of TM-DSC measurements consisted of three two-phase areas separated at a specific temperature at which three phases coexist. It was shown that the introduction of ionic comonomer (SA) into the structure of PNIPA results in a similar shape of phase diagram and differences are related to the shift of minimum critical points and intersections to the lower content of gel. In the contrast, it was shown that cross-linking of PNIPA makes the LCST of the PNIPA-BIS/water system become insensitive on changes of the gel content in sample.

The purpose of this paper is only paying attention to nontypical shape of well-known phase diagrams of PNIPA and its copolymers/water systems by TM DSC. Therefore a bare description of the thermoresponsive phase behavior of PNIPA gels without giving any molecular interpretation is only given. Detailed explanation and quantitative analysis of the described phenomena will be the subject of the next papers.

## Figures and Tables

**Table 1:** Molecular weights and molecular weight distributions of linear gels.

Gel	Mn [g/mole]	Mw [g/mole]	Mz [g/mole]	Molecular weight distribution *P* = Mw/Mn
PNIPA	343,200 (±0.3%)∗	731,800 (±0.3%)	1389,000 (±1%)	2.132 (±0.4%)
PNIPA-SA	505,700 (±0.3%)	1097,000 (±0.5%)	2274,000 (±2%)	2.169 (±0.6%)

∗Error in %.
